# Method for Assessing the Reliability of Molecular Diagnostics Based on Multiplexed SERS-Coded Nanoparticles

**DOI:** 10.1371/journal.pone.0062084

**Published:** 2013-04-19

**Authors:** Steven Y. Leigh, Madhura Som, Jonathan T. C. Liu

**Affiliations:** Stony Brook University (SUNY), Department of Biomedical Engineering, Stony Brook, New York, United States of America; University of California, Irvine, United States of America

## Abstract

Surface-enhanced Raman scattering (SERS) nanoparticles have been engineered to generate unique fingerprint spectra and are potentially useful as bright contrast agents for molecular diagnostics. One promising strategy for biomedical diagnostics and imaging is to functionalize various particle types (“flavors”), each emitting a unique spectral signature, to target a large multiplexed panel of molecular biomarkers. While SERS particles emit narrow spectral features that allow them to be easily separable under ideal conditions, the presence of competing noise sources and background signals such as detector noise, laser background, and autofluorescence confounds the reliability of demultiplexing algorithms. Results obtained during time-constrained *in vivo* imaging experiments may not be reproducible or accurate. Therefore, our goal is to provide experimentalists with a metric that may be monitored to enforce a desired bound on accuracy within a user-defined confidence level. We have defined a spectral reliability index (SRI), based on the output of a direct classical least-squares (DCLS) demultiplexing routine, which provides a measure of the reliability of the computed nanoparticle concentrations and ratios. We present simulations and experiments to demonstrate the feasibility of this strategy, which can potentially be utilized for a range of instruments and biomedical applications involving multiplexed SERS nanoparticles.

## Introduction

The field of biomedical optics has traditionally approached disease detection by deducing tissue status through the measurement of optical signals generated either intrinsically by tissue constituents [1]–[12] or extrinsically by targeted probes with known signatures [13]–[23]. In particular, diagnostic approaches involving both intrinsic and extrinsic Raman scattering have seen much success owing to the sharp separable features of Raman spectra. Intrinsic Raman spectroscopy has been demonstrated to identify malignant tissues with high sensitivity and specificity [2], [6], [8], [10] and has also enjoyed the benefit of expedited regulatory approval since no external contrast agent is necessary. [8], [9], [11], [12]. However, the spectral features of diagnostic value in intrinsic Raman detection are generated by highly conserved chemical constituents such as hydrocarbon, lipid, or nucleic acid bonds, which may be difficult to relate to pathological or clinical variables. Additionally, the acquisition time for intrinsic Raman spectroscopy is necessarily long given the low efficiency of Raman scattering and therefore presents a practical challenge for clinical diagnostics and imaging.

**Figure 1:**
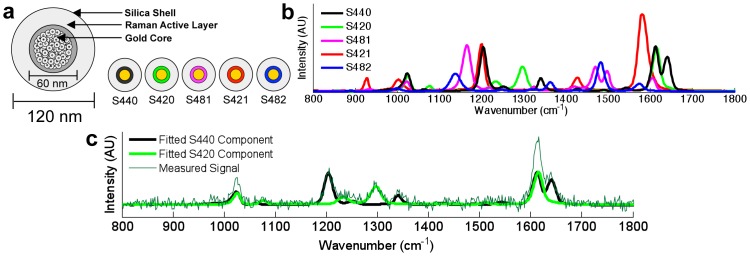
Surface-enhanced Raman scattering (SERS) nanoparticles. (a) Multiple flavors of nanoparticles exist where each nanoparticle contains a gold core coated with a Raman-active layer, encased in a silica shell. (b) Raman spectra of five nanoparticle flavors. (c) Example result from a least-squares routine showing the ability to demultiplex two different nanoparticles from a mixture under noisy conditions.

Recently, a number of groups [13]–[19], [21], [23] have explored the use of surface-enhanced Raman-scattering (SERS) nanoparticles that are engineered to emit bright distinct spectra (**Fig. 1**). These Raman-coded nanoparticles are available as different “flavors,” each emitting a characteristic spectral signature that may potentially be targeted against various molecular biomarkers for highly multiplexed molecular imaging. However, determining the reliability of particle concentrations or concentration ratios computed by spectral demultiplexing algorithms is a non-trivial challenge facing experimentalists since other signals such as detector noise, incomplete removal of excitation photons (laser background) and autofluorescence background degrade the accuracy of demultiplexing routines, especially as spectral overlap becomes a concern with increasing numbers of flavors competing within a limited spectral bandwidth.

Therefore, in this report, we propose a general method for quantifying the reliability of particle concentrations and ratios that are computed from a least-squares demultiplexing algorithm. First, this method is developed through numerical simulations of various mixtures of SERS nanoparticles in the context of noise and background signals. We define a metric, the spectral reliability index (SRI), which serves as a predictor of error in single- and multi-flavor applications. We further provide results from well-controlled experiments to assess the feasibility and accuracy of our approach. While initial experiments are intentionally simplified to verify the accuracy and reproducibility of these methods, our strategy could potentially be of value for a range of technologies that utilize targeted SERS-based nanoparticles to provide multiplexed measurements of molecular biomarkers both *in vitro* and *in vivo*.

## Methods

Since our ultimate goal is to provide experimentalists with a reliable measure of nanoparticle concentrations or concentration ratios, we set out to simulate spectral measurements of SERS nanoparticles under the varying noise and background conditions that may be encountered experimentally. Simulated spectra were generated first for single-flavor applications, then for two-particle applications where the relative concentrations between particle flavors ranged from 1∶1 to 5∶1, and finally for two-flavor ratios within three-flavor mixtures. The rationale for a 5∶1 maximum range of relative nanoparticle concentrations is based on the observation that signal contrast between tumor and normal tissues rarely extends beyond a factor of five for *in vivo* preclinical and clinical molecular imaging studies [14], [24], [25].

It bears mentioning that actual measurements of SERS nanoparticles in cells and tissues can include endogenous Raman background signals from tissues as well as variable autofluorescence and laser background components. With this in mind, our study makes a few assumptions that are often relevant for biomedical spectroscopy with SERS nanoparticles: 1) background signals are spectrally broadband and do not contain sharp narrowband spectral features that are morphologically similar to the spectral peaks generated by SERS nanoparticles (i.e., we assume that endogenous Raman signals are orders of magnitude weaker than the signals from SERS nanoparticles); 2) since the Stokes shift of Raman signals is higher than fluorescence signals, autofluorescence background signals are due to the slowly varying (broadband) tail at the long-wavelength side of the autofluorescence spectra; 3) a significant amount of stray laser light contributes to a broadband background at the CCD detector despite efforts taken to filter out the illuminating laser radiation (see section 2.5).

### 2.1. Spectral Simulations

In order to simulate a realistic spectral measurement, pure spectra from single- or multi-flavor particle mixtures were mixed with varying magnitudes of broadband background signals and zero-mean Gaussian-distributed white noise (Eq. (1)). Note that Eq. (1) incorporates all broadband background signals (e.g. laser background and autofluorescence background) into a single spectral component, *B*. Furthermore, Eq. (1) combines shot noise and other stochastic noise sources (e.g., detector readout noise and dark counts) into a single Gaussian-distributed noise term, denoted δ. See *Supplementary Material* for example source code (Source S1).
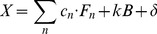
(1)
***X*** = simulated spectrum*c_n_* = concentration of SERS nanoparticle *n* ***F_n_*** = known reference spectrum of SERS nanoparticle flavor *n* *k* = scaling factor for background signal magnitude*B* = known reference spectrum of broadband background*δ* = noise

### 2.2. Demultiplexing SERS Spectra using DCLS

Other than sources of noise, it is assumed that each measured spectrum consists of a weighted sum of fixed nanoparticle spectra (*F_n_*) and broadband background signals (*B*). Based on the assumption that the combination is linear, we employ a linear least-squares algorithm in MATLAB (MathWorks) to compute the relative nanoparticle weights (*w_n_*). A third-order polynomial is included to account for broadband background signals that are not captured by the broadband noise reference spectrum (*B*) [26]. See *Supplementary Material* for example source code (Source S2).
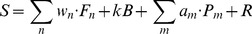
(2)
***S*** = measured spectral data*w_n_* = weight of SERS flavor *n* ***F_n_*** = known reference spectrum of SERS nanoparticle flavor *n* *k* = scaling factor for background signal magnitude*B* = known reference spectrum of broadband background*a_m_* = weight of *m* ^th^-order polynomial term***P_m_*** = *m* ^th^-order polynomial term (for baseline correction)***R*** = residual (minimized by least-squares algorithm)

### 2.3. Quality Metrics

Below we describe metrics called ‘relative fitting error’ (RFE) and ‘spectral reliability index’ (SRI). RFE and SRI are “goodness-of-fit” metrics that quantify how well the simulated spectra can be decomposed into the reference spectra defined *a priori*.

A common approach to quantify the reliability of a spectral fitting routine is to compare the norm of the fit to the norm of the input signal, a metric that has previously been termed the relative fitting error, RFE, given in Eq. (3) [27]. An RFE of 1 indicates a perfect fit and approaches zero as the fit degrades (i.e. the optimal fit lacks the ability to represent the input signal as a linearly weighted sum of the references supplied). However, in cases where the particle signals are weak in comparison to the broadband background signals, the reported RFE may be biased towards 1 due to the fact that the background and baseline signals are fit with high fidelity even if the fit for the nanoparticle components is poor. We therefore modify the RFE metric to ignore non-particle components so that they do not contribute to the apparent reliability of the least-squares fit. We call this modified measure the spectral reliability index (SRI), as shown in Eq. (4).
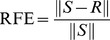
(3)

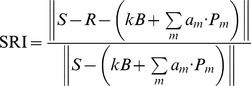
(4)


Although both RFE and SRI are informative consolidated measures of goodness of fit, the biomedical application of these fitting algorithms requires us to examine errors in the actual nanoparticle weights computed. For single-particle cases, we use the standard percent error definition. In cases with more than one particle, we compute each individual particle’s error and generate a single value that we call the composite error for the entire mixture (Eq. (5)). This will be discussed further in section 2.4.
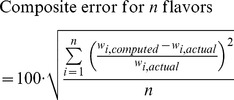
(5)


**Figure 2:**
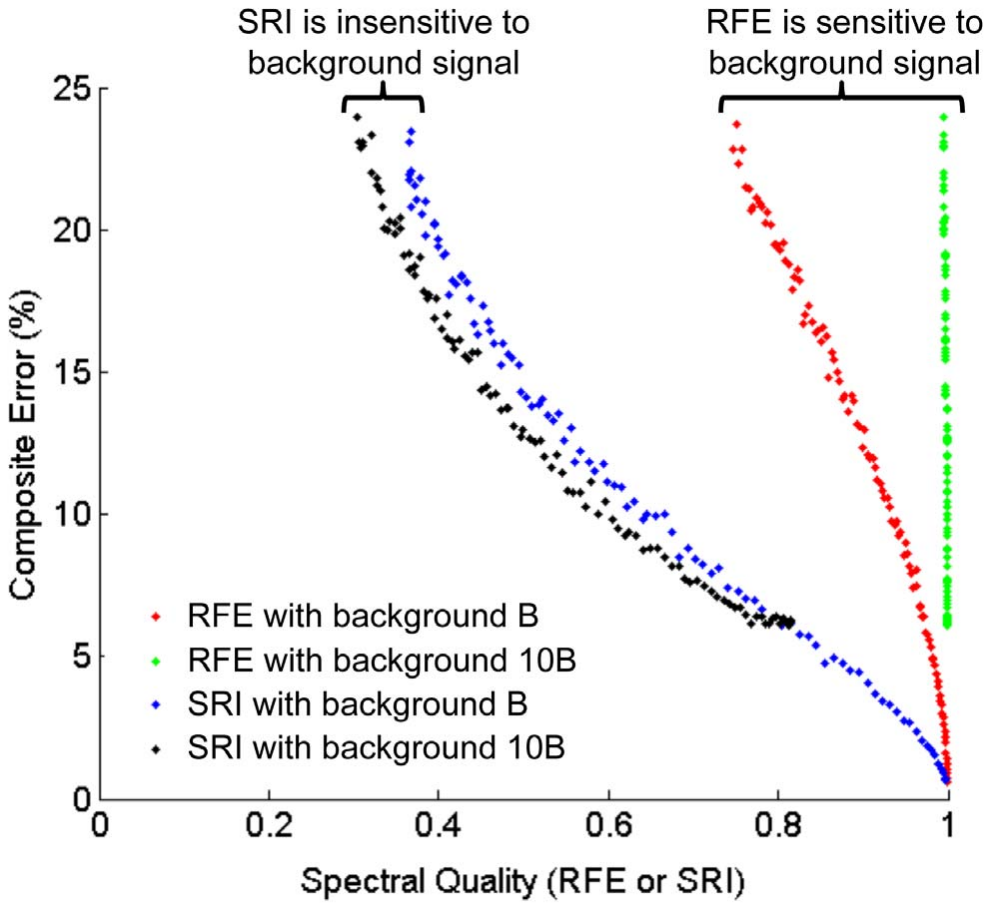
Quality Metrics. Simulations of composite error as a function of two different spectral quality metrics, RFE (Eq. (3)) and SRI (Eq. (4)). Note that the RFE metric is highly sensitive to the broadband background level (*B* vs. 10*B*) whereas the SRI metric is relatively insensitive to these variations in background and provides a better indication of the “goodness-of-fit” for the SERS nanoparticles themselves.

An example of the difference between RFE and SRI is shown in **Fig. 2**. In this example, varying levels of noise, *δ*, are superimposed on a fixed-magnitude spectrum representing the pure Raman signals from two SERS nanoparticle flavors. In addition, these simulated spectra contain either a low magnitude of a broadband background, *B,* or an order-of-magnitude higher level of background, 10*B*. Increasing levels of noise (*δ*) in the simulated spectra result in degraded spectral quality and lower levels of the quality metrics, RFE and SRI. However, the simulation demonstrates that SRI is relatively insensitive to the magnitude of the broadband background. In contrast, the RFE is highly sensitive to the background level and is biased towards 1 when this background dominates the signal. In other words, the RFE does not provide a good indication of the “goodness-of-fit” of the SERS particles themselves. Rather, the RFE indicates a “goodness-of-fit” for a spectrum as a whole, which can be dominated by background components instead of the components of interest (SERS nanoparticles).

### 2.4. Measurement Error and Confidence

In an ideal situation, an experimentalist would obtain a single measurement and assume that the nanoparticle weights computed by a DCLS routine are accurate and reproducible in subsequent measurements. In reality, the randomness of the noise and broadband background signals results in a distribution of measurement errors. Thus, the goal of our simulations is to provide researchers with a minimum necessary SRI value to guarantee a maximum bound on concentration error (e.g., 10% error for any single measurement). To achieve this, a measure of confidence must be assigned to the maximum bound on concentration error that is desired for a single measurement. We first note that the concentration errors generated by the DCLS routine are normally distributed (Gaussian) for any single nanoparticle flavor. Second, we define a composite error for multi-flavor mixtures, which is the root-mean-squared error of all nanoparticle flavors (Eq. (5)). Defining the composite error in this way implies that the composite error for a mixture of nanoparticle flavors (*n* >1) is described by a gamma distribution [28].

In order to assign a level of confidence to the error of a single measurement that an experimentalist may perform, we simulate 50,000 spectral measurements corresponding to a particular SRI, process them through a DCLS demultiplexing routine, and then construct a histogram of concentrations errors (results shown later in section 3.2). After fitting a Gaussian (*n*  = 1 flavor) or gamma (*n* >1 flavor) distribution to this histogram of concentration errors, the error value, *e*_80_, corresponding to a certain percentile (in our case, 80%) of the cumulative distribution function is found. In other words, since 80% of the possible error values corresponding to a particular SRI are less than this error value, *e*_80_, an experimentalist who has taken a single measurement and obtained an SRI value from the DCLS routine can be 80% confident that the error in their measurement is at most *e*_80_ (results shown later in section 3.2).

### 2.5. In Vitro Experiments

**Figure 3:**
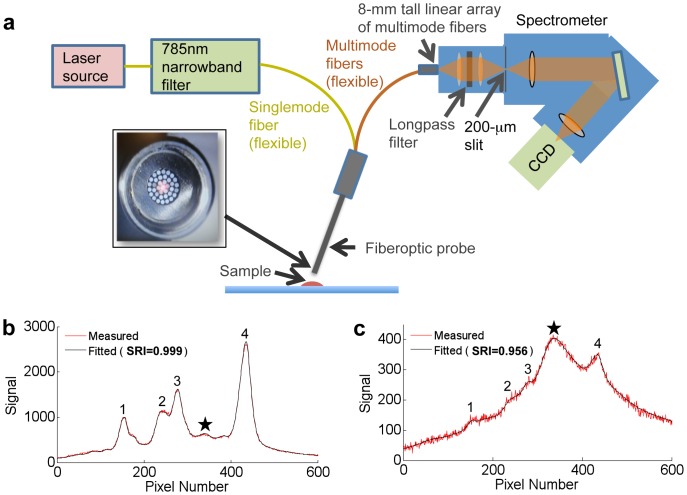
Measurement apparatus. (a) A spectrometer with CCD detector is used to capture Raman signals from a nanoparticle sample illuminated with a 785-nm laser source. See text for details. (b) Example of a strong signal with a high SRI and (c) a weak signal with a lower SRI, in which noise and broadband background signals increasingly dominate over the SERS signals. Representative SERS peaks are numbered 1–4 and the peak of the broadband background is labeled with a star.

Various flavors of SERS nanoparticles were obtained from Cabot Corporation, formerly Oxonica Materials (Mountain View, CA). Droplets (5 µL) of varying nanoparticle concentrations and mixtures between 0.8 and 800 pM were placed on a glass slide and point measurements were obtained using a custom fiberoptic probe (Fiberguide Industries, Stirling, NJ) that is angled at ∼45 degrees to the glass slide to minimize specular reflections. On the illumination side of the device, light from a 785-nm diode laser (30 mW) is first filtered with a narrow-bandpass filter (Semrock, LD01-785) prior to being coupled into the illumination fiber at the center of the fiber bundle. This removes off-resonant laser noise (including amplified spontaneous emission) that may be collected by the multimode fibers and contribute background signal and shot noise to the Raman spectra. Raman signals are collected via a bundle of 36 close-packed multimode fibers (200-µm core) surrounding one singlemode illumination fiber (**Fig. 3a**). These multimode fibers are reconfigured into an 8-mm tall linear array at the proximal end of the bundle, which serves as the entrance slit into a spectrometer.

Experiments are performed with a custom spectrometer from Bayspec (RamSpec NIR) outfitted with a cooled deep-depletion spectroscopic CCD (Andor DU920P-BR-DD). The Bayspec spectrograph utilizes low-f/# optics (f/1.8 or NA  = 0.28) to effectively image Raman signals from the multimode collection fibers (NA  = 0.21) onto a proprietary volume phase grating and then onto the Andor CCD. The Andor DU920P CCD contains 1024 x 256 pixels, with a 6.2-mm height (256-pixel dimension) that is exactly matched to the height of the image of our linear fiber bundle array with 4∶3 de-magnified imaging between the entrance slit and the CCD detector (the NA increases from 0.21 to 0.28). Since a large amount of the laser light (785-nm illumination wavelength) is collected by our multimode collection fibers, a relay extension is built into the front of the spectrograph to allow for the placement of a longpass interference filter (Semrock LP02-830RU-25) to reject illumination light at 785-nm, as well as any autofluorescence background at shorter wavelengths than the Raman peaks (<830-nm). This 4∶3 demagnification relay extension contains a 150-µm slit on the far end where the image of the linear array of fibers is refocused. This slit spatially filters out the diffuse stray light in the relay chamber from photons that are rejected by the longpass filter (**Fig. 3a**). However, this spatial filter is not perfect and still allows a significant amount of stray laser light into the spectrometer, which contributes to the broadband background seen by the CCD.

The reference spectra of the nanoparticles (*F_n_*) as well as the background reference spectrum (*B*) are background-corrected to remove detector offset due to readout noise. Spectra are acquired through full-vertical binning of the pixels in each column of the CCD using the full dynamic range of the CCD sensor to minimize digitization noise. For experimental validation of simulations, particle concentrations are varied to monitor the effect of background and detector noise on the accuracy of demultiplexing. Note that at our measurement conditions (full vertical binning at 100 ms integration times), using a cooled detector at −65 deg C, detector readout noise dominates over dark counts (thermal noise). Deeper cooling may be necessary to suppress dark counts at longer integration times, at the expense of a slightly reduced quantum efficiency in the near infrared. **Figs. 3b and 3c** show two example spectra obtained experimentally at high and low particle concentrations, respectively, along with the least-squares fitted spectra (*S*). Note that in the low-concentration condition shown in **Fig. 3c**, the spectral features from the SERS nanoparticles are increasingly obscured by the broadband background and noise (detector readout noise and shot noise).

## Results and Discussion

### 3.1. Single-flavor Concentrations: Simulations and Experiments

**Figure 4:**
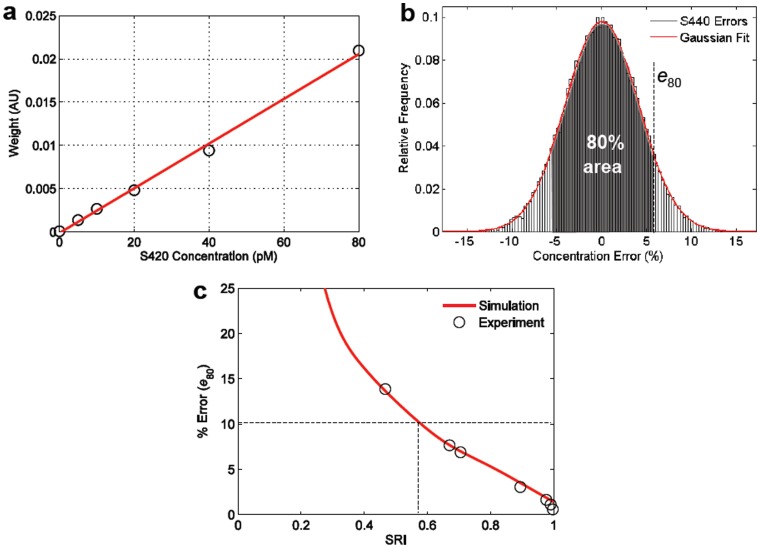
Spectral quality characterization of single-flavor samples. (a) Weights returned by the least-squares routine are linear over the range of measured concentrations. (b) Errors from multiple measurements of the same single-flavor sample are normally distributed. Shaded region indicates where 80% of the errors lie. (c) A plot of error vs. SRI, in which the reported error is the 80% confidence bound (*e*_80_). The simulations agree well with the results found experimentally.

Experiments were first conducted with single nanoparticle flavors at various concentrations. **Fig. 4a** demonstrates the linearity of these measurements. **Fig. 4b** shows the normal distribution of errors for 5000 experimental measurements at a specific particle concentration. The shaded region indicates the range within which 80% of the errors lie (*e*_80_). Finally, **Fig. 4c** shows how the percent error (at 80% confidence; *e*_80_) varies as a function of SRI. A comparison between simulations and experiments (**Fig. 4c**) reveals excellent agreement and demonstrates the predictive power of the simulations. We find that for any single measurement in this particular experiment, spectral data with an SRI of 0.57 is necessary to guarantee an error ≤10% with 80% confidence.

### 3.2. Dual-flavor Concentrations: Simulations and Experiments

**Figure 5:**
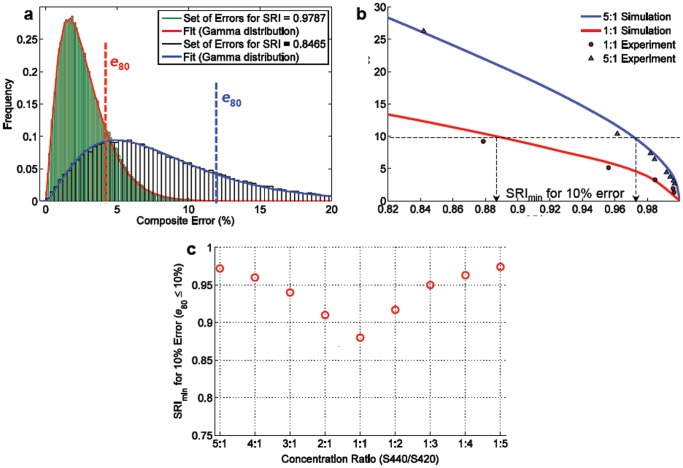
Spectral quality characterization of two-flavor mixtures. (a) Composite errors for multi-particle mixtures are gamma-distributed. Vertical lines indicate bounds for the 80^th^ percentile of error values (*e*_80_) that may occur for a particular SRI. (b) A plot of error (*e*_80_) vs. SRI for a 1∶1 mixture of particle flavors and a 5∶1 mixture of flavors. Simulations (solid lines) closely predict experimental results. (c) A plot of the minimum SRI required to ensure a composite error ≤10% with 80% confidence. This dual-flavor example shows how the minimum SRI value depends upon the mixture ratio.

Next, a dual-flavor mixture is analyzed through simulations and experiments (**Fig. 5**). In **Fig. 5a**, simulated histograms are shown of composite errors for a dual-flavor mixture of nanoparticles. Note that the composite-error histograms obey a gamma distribution, as described previously, and that we choose to use the 80^th^ percentile (80% confidence level) for our analyses (*e*_80_). By constructing composite-error histograms (e.g. **Fig. 5a**) over a range of SRI conditions, we calculate how composite error varies as a function of SRI. In **Fig. 5b**, both a 1∶1 equimolar mixture as well as a 5∶1 mixture of particle flavors is simulated and experimentally validated at a range of concentrations (and subsequently a range of SRI values). Note that a higher SRI is required for a 5∶1 mixture, compared to a 1∶1 mixture, to guarantee an identical requirement for error of ≤10% with 80% confidence. This is due to the fact that in the 5∶1 case, although the lower-concentration flavor is fit with less fidelity than the higher-concentration flavor, the latter component dominates the overall spectrum and therefore dominates the SRI value as well. **Fig. 5c** is a plot (simulation) of the minimum SRI that is required to guarantee a composite error of ≤10% with 80% confidence, over a range of mixture ratios between two particle flavors (from 1∶5 to 5∶1). This plot shows how the minimum required SRI depends on the mixture ratio. In other words, in order to calculate the minimum SRI necessary to ensure a desired level of accuracy, one must also take into account the relative ratio of the particle flavors. This will be further elaborated in section 4 of this paper.

### 3.3. Triple-flavor Ratios: Simulations and Experiments

**Figure 6:**
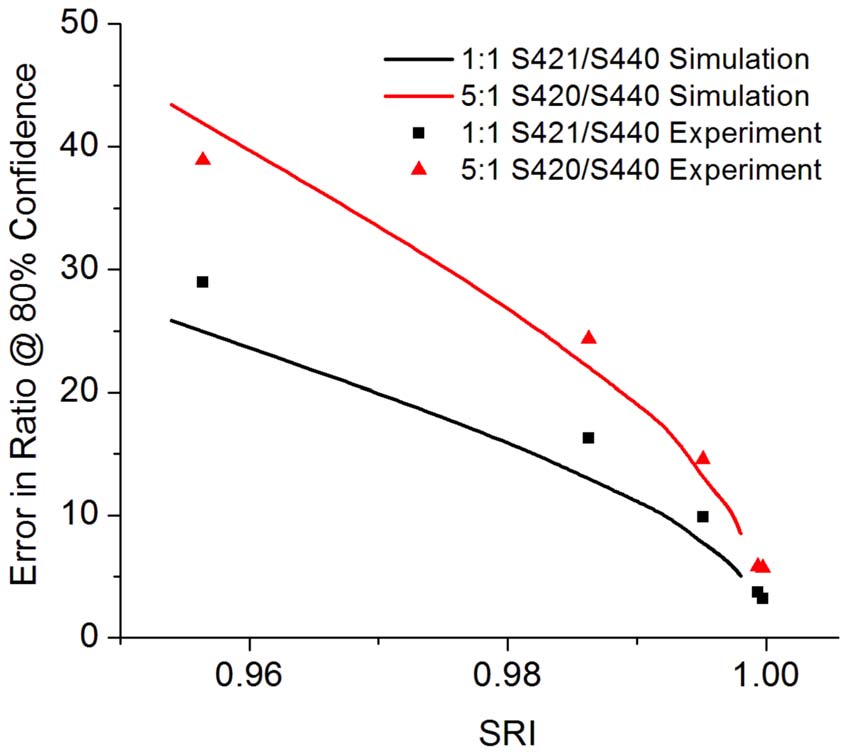
Spectral quality characterization of three-flavor mixtures. The plot shows percent error (*e*_80_) in the ratio between particle flavors as a function of SRI.

Finally, a triple-flavor mixture is investigated via simulations and experiments, and the error in the concentration ratios is presented (**Fig. 6**). In practice, the ratio between various nanoparticle flavors is likely to be more important than absolute concentrations for *in vivo* molecular imaging studies. This is due to the fact that with both topical and systemic delivery, absolute particle concentrations are affected by a host of nonspecific effects, such as uneven particle delivery and washout of unbound probes, non-chemical enhanced-permeability-and-retention (EPR) effects, nonspecific chemical binding, as well as variations in optical alignment and working distance. Ratiometric measurements of particles targeted against various biomarkers that are referenced against nontargeted control particles offer the ability to quantify specific vs. nonspecific binding in spite of these confounding factors [29] as well as to quantify binding potentials [30]. Therefore, in a multiplexing application involving *n* targets, it is typically desirable to include an untargeted (nonspecific) negative control agent to serve as a single reference for the *n* targeted particles. **Figure 6** demonstrates a three-particle simulation and experiment where one particle in the mixture (S440) is assumed to be a negative control for a weak target (S421) as well as a strong target (S420). The weak target does not exhibit elevated expression relative to the negative control (1∶1 ratio) whereas the strong target exhibits a 5-fold overexpression relative to the negative control.

## Summary and Conclusions

**Figure 7:**
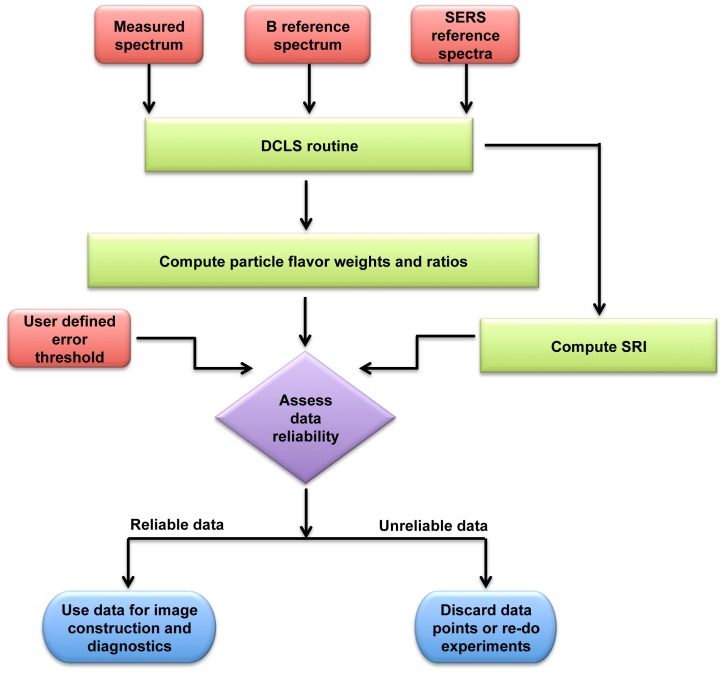
Algorithm summary. A flowchart illustrating how SRI may be used in practice to ensure that a SERS-based multiplexed molecular diagnostic is reliable. See text for details.

In summary, we have presented an analysis of the accuracy of least-squares demultiplexing for spectral measurements of SERS particle mixtures. In particular, we have proposed and demonstrated the feasibility of a spectral reliability index (SRI) that correlates with measurement accuracy. The flowchart shown in **Fig. 7** illustrates how the SRI may be employed to ensure desired measurement accuracy in a multiplexed molecular imaging study with SERS nanoparticles. The required inputs for this reliability analysis are: 1) raw spectra acquired from a Raman detection probe; 2) reference spectra (*F_n_*) of nanoparticle flavors; 3) reference spectrum (*B*) of the background; and 4) a user-defined error threshold (a maximum error at a defined confidence level). A DCLS fitting algorithm takes the first 3 inputs listed above and computes a number of parameters, including the weights (*w_n_*) of the SERS particle flavors and the ratios between flavors. The results of the DCLS algorithm are also used to calculate an SRI for each spectral measurement according to Eq. (4). The main decision point depicted in the flowchart (purple diamond) involves taking the computed SRI, along with the computed particle ratios, to determine if the data satisfies the user-defined reliability criteria. Note that both the SRI and the particle ratios are needed to make this determination, as shown earlier in **Fig. 5c**, since measurement accuracy is a function of both.

In practice, a set of look-up tables could be generated through simulations in order to establish the relationships between error vs. SRI and particle ratios. This would allow one to rapidly assess the reliability of spectral demultiplexing measurements in real time. These simulations and look-up tables would be different for every device, experimental application (e.g., number of particle flavors), and user-defined error threshold since the relationship between error and SRI depends upon all of these instrument- and application-dependent factors. However, the general algorithm depicted in the flow chart in **Fig. 7** applies for a range of applications utilizing SERS-coded nanoparticles for multiplexed molecular diagnostics. If spectral data do not satisfy the user-defined reliability criteria, the data may be discarded or the experiments may need to be repeated to improve the quality of the data, possibly through averaging or by changing system parameters (e.g., detector integration times, laser powers, etc.).

As SERS-coded nanoparticles gain popularity in animal studies and eventually for clinical diagnostics, the reliability of demultiplexing algorithms will need to be constantly assessed. Although spectroscopy is powerful in its ability to convey large amounts of information, performing accurate spectroscopic measurements is far from trivial. It has been our intent to develop a general metric and algorithm for rapidly assessing the experimental accuracy of spectral measurements involving multiplexed SERS nanoparticles. While we have performed simulations and experiments to demonstrate the basic feasibility of this approach using well-controlled experimental conditions under the simplifying assumptions mentioned in section 2, future studies are needed to demonstrate the accuracy and utility of these methods in a variety of biomedical applications such as *in vitro* diagnostics or *in vivo* molecular imaging.

## Supporting Information

Source S1 **Simulating acquired spectra.**
(M) Click here for additional data file.

Source S2 **Demultiplexing experimentally-acquired spectra.**
(M) Click here for additional data file.
